# A comparison between real-time intraoperative voice dictation and the operative report in laparoscopic cholecystectomy: a multicenter prospective observational study

**DOI:** 10.1007/s00423-023-03079-w

**Published:** 2023-08-25

**Authors:** Özgür Eryigit, Floyd W. van de Graaf, Vincent B. Nieuwenhuijs, Meindert N. Sosef, Eelco J. R. de Graaf, Anand G. Menon, Marilyne M. Lange, Johan F. Lange

**Affiliations:** 1 Department of Surgery, Erasmus University Medical Center, Internal Postal Address H-173, P.O. Box 2040, 3000 CA Rotterdam, the Netherlands; 2 Department of Surgery, Isala, Zwolle, the Netherlands; 3 Department of Surgery, Zuyderland Medical Center, Sittard-Geleen and Heerlen, the Netherlands; 4 Department of Surgery, IJsselland Hospital, Capelle Aan Den IJssel, the Netherlands; 5 Department of Pathology, Amsterdam University Medical Center, Location VUmc, Amsterdam, the Netherlands

**Keywords:** Patient safety, Voice dictation, Operative reporting, Laparoscopic cholecystectomy

## Abstract

**Purpose:**

The current operative report often inadequately reflects events occurring during laparoscopic cholecystectomy (LC). The addition of intraoperative video recording to the operative report has already proven to add important information. It was hypothesized that real-time intraoperative voice dictation (RIVD) can provide an equal or more complete overview of the operative procedure compared to the narrative operative report (NR) produced postoperatively.

**Methods:**

SONAR is a multicenter prospective observational trial, conducted at four surgical centers in the Netherlands. Elective LCs of patients aged 18 years and older were included. Participating surgeons were requested to dictate the essential steps of LC during surgery. RIVDs and NRs were reviewed according to the stepwise LC guideline of the Dutch Society for Surgery. The cumulative adequacy rates for RIVDs were compared with those of the postoperatively written NR.

**Results:**

79 of 90 cases were eligible for inclusion and available for further analysis. RIVD resulted in a significantly higher adequacy rate compared to NR for the circumferential dissection of the cystic duct and artery (NR 32.5% vs. RIVD 61.0%, *P* = 0.016). NR had higher adequacy rates in reporting the transection of the cystic duct (NR 100% vs. RIVD 77.9%, *P* =  < 0.001) and the removal of the gallbladder from the liver bed (NR 98.7% vs. RIVD 68.8%, *P* < 0.001). The total adequacy was not significantly different between the two reporting methods (NR 78.0% vs. RIVD 76.4%, *P* = 1.00).

**Conclusion:**

Overall, the adequacy of RIVD is comparable to the postoperatively written NR in reporting surgical steps in LC. However, the most essential surgical step, the circumferential dissection of the cystic duct and artery, was reported more adequately in RIVD.

**Supplementary Information:**

The online version contains supplementary material available at 10.1007/s00423-023-03079-w.

## Introduction

In the past century, the narrative operative report (NR) has been the mainstay of surgical procedure documentation.

Either written, dictated and then described, or typed directly in the electronic patient file, it provides a narrative in which the course of the surgical procedure is described. Despite its long use, the traditional NR is lacking in objectivity by default and portrays a subjective view of the surgeon by definition, therefore often omitting or even inaccurately reflecting essential procedural information [1]. In the case of laparoscopic cholecystectomy (LC), prior research has demonstrated that the current form of NR is not sufficient to adequately record the critical view of safety (CVS), in which the cystic duct and artery are circumferentially identified in the limitations of Calot’s hepatobiliary triangle, prior to transection [2]. This step is of great importance to perform correctly, but also to document in an adequate fashion, because 70–80% of iatrogenic bile duct injuries (BDI) originate during this step due to misidentification of biliary structures [3, 4]. Also, with BDI potentially leading to life threatening complications, prolonged hospitalization, high financial expenditures, and risk of litigation, [5] it is warranted that proper documentation exists.

Several methods to improve the documentation of CVS, such as photography and video recording, have been investigated and proven feasible as an adjunct to NR [2, 6–9]. In a recently published practice guideline on prevention of BDI during LC, CVS was recommended by an expert panel as anatomical recognition method. This panel also agreed on the superiority of video documentation to operative reports for the accurate documentation of CVS [10]. In the Netherlands, it is standard practice to capture CVS either with endoscopic screenshots or to a much lesser extent with video recording [11–13]. This method, however, is not widely implemented in the rest of the world.

In a Dutch cross-disciplinary survey among surgeons, gynecologists, urologists, and corresponding residents in training, half of the respondents agree with the fact that the currently used narrative operative report is insufficient. Although audio recording is still an unknown territory for many physicians, already 32.7% of respondents recognized the added value of video and audio recordings and 39.6% of respondents saw potential in the use of this modality for quality control purposes. The respondents also found video and audio recordings useful for educational purposes (61.7%), for proctoring (46%), as a supportive role in medicolegal proceedings (41.5%), and as information for patients, family and/or colleagues (21.8%) [14].

Despite the benefits and increasing availability of audio recording modalities in the operating room, current videos of LCs are recorded without sound, potentially withholding a better understanding of the intraoperative proceedings. To further broaden the range of alternatives to NR and to investigate the feasibility of a real-time dictated operative report compared to NR, produced with delay, we intended to introduce real-time intraoperative voice dictation (RIVD) during LC. In this study, our aim is to focus on RIVD, to investigate whether this reporting modality can provide an equal or better understanding of LC compared to the traditional NR. To our knowledge, no study has been conducted yet in which the availability of information essential to the surgical procedure has been compared between an intraoperatively voice dictated report and a postoperatively written report.

## Material and methods

This study is part of the Simultaneous Video and Audio Recording of Laparoscopic Cholecystectomy Procedures (SONAR) trial, which is a multicenter prospective observational study conducted at four surgical centers (Isala, Zuyderland Medical Center, IJsselland Hospital, and Park Medical Center) in the Netherlands between 18 September 2018 and 13 November 2018. The medical research and ethics committee of the Erasmus University Medical Center exempted this study from the Research Involving Human Subjects Act and Institutional review boards of the participating centers provided separate approval of this trial prior to local initiation. Written informed consent was obtained from the operators for the use of their voice recordings. This study has been reported in line with the Standards for Quality Improvement Reporting Excellence (SQUIRE) criteria.

### Study subjects

Operators (surgeons, fellows, and surgical residents) from the respective institutions were approached for participation. Before the surgical procedure, the operator was requested to dictate their surgical steps and considerations in real-time over the course of the surgical procedure with a wireless and wearable microphone (Revolabs Xtag™ Wireless Microphone, Yamaha Unified Communications Inc, Sudbury, MA, U.S.A.). The microphone was attached to the operator’s scrub top. RIVDs were saved as music player 3 (MP3) files using Audacity® recording and editing software version 2.3.3. (The Audacity Team) on a password-protected external hard drive. Images depicting the wireless microphone attached to the operator's scrub top and the microphone placed in its charger base have been included as supplementary materials for visual reference. After the completion of each surgical procedure, the NR was reported by the same operator. Elective LCs of patients aged 18 years or older were eligible for inclusion. Study cases with incomplete RIVDs or unavailable NRs were excluded.

### Data collection

The audio recordings were initiated at the moment of endoscope introduction in the abdomen and terminated upon disconnection of the endoscope. RIVDs and NRs were retrieved and subsequently anonymized for further analysis and comparison. During this study, RIVDs were not entered as part of the medical record, as they were merely being used for quality control purposes. Patient data regarding baseline characteristics were retrieved from the patients’ electronic health records and anonymously entered into a database.

### Study outcome

**Fig. 1:**
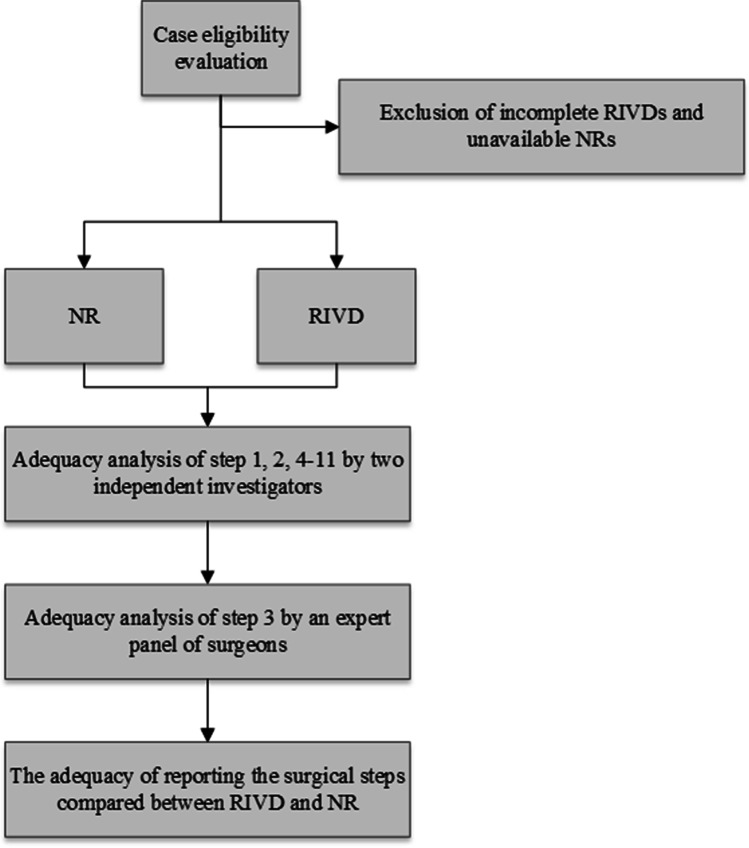
Flow diagram summarizing the execution of this study

The recorded audio, as well as the corresponding NRs, were reviewed for adequacy according to predefined key steps for LC, as mentioned in Appendix A. Adequacy was defined as the competent depiction of a surgical step. Recordings were analyzed by two researchers (ÖE, FvdG) based on the stepwise LC guideline of the Dutch Society for Surgery [11]. The independent reviewer form is shown in the Supplement (Form 1). Subsequently, steps regarding the circumferential dissection of the cystic duct and artery were analyzed by an expert panel of two surgeons qualified in laparoscopic surgery (JL, AM) for an adequate depiction in both RIVD and NR. In accordance with the previously mentioned guideline provided by the Dutch Society for Surgery, the circumferential dissection of the cystic duct and artery can only be performed after full mobilization of the gallbladder's neck through the dissection of approximately one-third of the distal part of the gallbladder from the liver bed has been obtained. The cumulative adequacy ratings for RIVD were compared with those for NR. A flow diagram summarizing the execution of this study is shown in the Supplement (Fig. 1).

### Statistical analysis

Categorical data are presented as numbers and percentages, normally distributed data are expressed as median (interquartile range) or mean (standard deviation). RIVDs and NRs were individually compared with the assumption that a specific aspect of the procedure was identical for both RIVD and NR. Adequacy between individual steps were compared with the exact McNemar’s test [15]. The total adequacy was compared with the paired samples t-test or Wilcoxon signed-rank test, depending on normality. In case of multiple comparisons, Bonferroni correction was applied by multiplying the obtained *P* values with the number of completed tests. A *P* value of < 0.05 was considered statistically significant. Data were analyzed with statistical software R, version 3.4.1, for Windows (http://www.r-project.org). Flow diagrams were created with Microsoft Visio version 8.1.1 for Windows (GraphPad Software, La Jolla, CA, U.S.A.).

### Sample size

The sample size calculation was based on prior data by Wauben et al., evaluating the quality of NR [1]. For this calculation, CVS was selected as most essential step, for this is unequivocally the most critical part of the surgical procedure, thus most important to report adequately. In 79.2% of the video recordings, CVS was observed. In 50.4% of the reviewed cases CVS was adequately reported in NR and observed in the video recordings. A minimal sample size of 73 procedures was calculated with α = 0.05, power = 0.80, and δ equal to 0.10. In this trial, 90 patients were intended to be included after accounting for loss of data. No prior trials were found in which audio recordings were used during surgery for operative reporting.

## Results

### Study population

Between 18 September 2018 and 13 November 2018, 90 patients who met the inclusion criteria underwent LC in the participating centers. Subsequently, 11 cases were excluded from the analysis, 10 due to technical malfunctioning of the recording equipment or problems in data storage and one because of early termination of the surgical procedure due to suspected liver metastases.79 RIVDs and NRs of the SONAR-trial were eligible for inclusion and available for further analysis. 49 of 79 patients were women (62.0%) and the mean (SD) age was 54.3 (15.9) years. Patient and surgery characteristics are presented in Tables 1 and 2. Twenty-four different primary operators conducted the procedures, with a mean number of 3 cases per operator (range, 1–18). Two procedures were converted to open LC due to difficulties with identifying the anatomical structures.

**Table 1:** Patient Characteristics

Characteristic	Mean (SD)
Total included, No	79
Excluded, No. (%)	11 (12.2)
Age, y	54.3 (15.9)
Women, No. (%)	49 (62.0)
BMI	28.9 (5.2)

Abbreviation: *BMI*, body mass index (calculated as weight in kilograms divided by height in meters squared)

**Table 2:** Surgery Characteristics

Characteristic	Operations (*n* = 79)
Primary operator function, No. (%)	
Surgeon	9 (11.4)
Fellow	59 (74.7)
Surgical resident	11 (13.9)
Secondary operator function, No. (%)	
Surgeon	20 (25.3)
Fellow	11 (13.9)
Surgical resident	2 (2.5)
Operation assistant	37 (46.8)
Medical student	9 (11.4)
Surgery duration, mean (SD), min:s	43:21 (24:52)
Indication for surgery, No. (%)	
Symptomatic cholelithiasis	66 (83.5)
Other	8 (10.1)
Acute cholecystitis	5 (6.3)
Time > 7 d between onset acute cholecystitis and surgery, No./total No. (%)	5 (100.0)
Conversion to open surgery, No. (%)	2 (2.5)

### Quantitative technical data

A total of 65 h 7 min of audio footage was recorded. The mean (SD) duration per recording was 49 min (25). The total required digital storage space was 2 851 megabytes, with a mean (SD) size of 36 (24) megabytes per case.

### Adequacy

Adequacy rates are summarized in Table 3. After Bonferroni correction, RIVD resulted in a significantly higher adequacy rate compared to NR for the circumferential dissection of the cystic duct and artery (NR 32.5% vs. RIVD 61.0%, *P* = 0.016). NR had a higher adequacy rate in reporting the transection of the cystic duct (NR 100% vs. RIVD 77.9%, *P* = 0.00026) and the removal of the gallbladder from the liver bed (NR 98.7% vs. RIVD 68.8%, *P* < 0.0001). The total adequacy was not significantly different between the two reporting methods (NR 78.0% vs. RIVD 76.4%, *P* = 1.00).

**Table 3:** Adequacy rates for NR and RIVD

No./total No. of steps (%)			
Procedure steps (*N* = 79 operations)	NR	RIVD	*P* value for exact McNemar’s test^a^
1a. Introduction of the first accessory trocar	79/79 (100.0)	72/79 (91.1)	0.266
1b. Introduction of the second accessory trocar	79/79 (100.0)	72/79 (91.1)	0.266
1c. Introduction of the third accessory trocar	79/79 (100.0)	72/79 (91.1)	0.266
2a. Inspection of the gallbladder	39/79 (49.4)	46/79 (58.2)	1.00
2b. Inspection of the liver condition	17/79 (21.5)	33/79 (41.8)	0.12
3. Circumferential dissection of the cystic duct and artery	25/77 (32.5)	47/77 (61.0)	0.016
4. Transection of the cystic artery	71/77 (92.2)	64/77 (83.1)	1.00
5. Transection of the cystic duct	77/77 (100.0)	60/77 (77.9)	0.00026
6. Removal of the gallbladder from the liver bed	76/77 (98.7)	53/77 (68.8)	< 0.0001
7. Inspection of liver hemostasis	65/77 (84.4)	56/77 (72.7)	1.00
8. Presence of spill	32/35 (91.4)	33/35 (94.3)	1.00
9. Saline irrigation	27/34 (79.4)	32/34 (94.1)	1.00
10. Drain placement	3/3 (100.0)	3/3 (100.0)	1.00
11a. Removal of the first accessory trocar	60/79 (75.9)	63/79 (79.7)	1.00
11b. Removal of the second accessory trocar	60/79 (75.9)	64/79 (81.0)	1.00
11c. Removal of the third accessory trocar	60/79 (75.9)	62/79 (78.5)	1.00
Total	849/1089 (78.0)	832/1089 (76.4)	1.00^b^

^a^Bonferroni corrected

^b^Wilcoxon signed rank test (Bonferroni corrected)

## Discussion

As the availability of modalities to capture events that transpire during surgery is increasing, the call for improvement in surgical reporting will become increasingly evident. However, in surgical specialties, the operative report has remained unaltered in the last decades.

RIVD as one of these modalities might be of benefit, as it could provide a real-time narrative of the course of surgery, including comments on certain important findings that may not be included in the traditional NR.

This study demonstrates that, overall, RIVD during LC is comparable to NR in the adequate depiction of essential surgical steps. However, the circumferential dissection of the cystic duct and artery, the most essential step in LC, was reported significantly more accurately in RIVD compared to NR.

In NR, 78.0% of the essential steps were reported according to the guidelines. However, for quality control purposes, the adequacy of NR in its current form is still insufficient: the lowest adequacy rate for NR was the inspection of the liver condition (21.5%), the circumferential dissection of the cystic duct and artery (32.5%), and the inspection of the gallbladder condition (49.4%). The inadequate description of the inspection of the gallbladder and the liver conditions might be caused by the fact that operators are less likely to report normal organ conditions. Though, underreporting will impede future readers of NR in ascertaining the absence of any atypical findings. The circumferential dissection phase is reported inadequately in NR mainly due to the fact that most operators only mention *‘Calot’s triangle’*, *‘dissection of the cystic duct and artery’*, or just simply *‘CVS’*. Earlier findings by van de Graaf et al. demonstrated that many operators are unacquainted with the correct definition of CVS [16]. In this respect, we believe that the description of this step should at least contain keywords describing the circumferential dissection of the cystic duct and artery. Possible reasons for inaccuracy in NR relate partly to practical problems. It was common in the participating centers that multiple LCs were performed in close succession. Subsequently, reporting several, nearly identical, procedures at the end of the day may lead to inaccuracies due to physical and mental fatigue and tiredness. Moreover, the adequacy could also be variable dependent on years of work experience. Some operators used self-made formats to quickly fill in NRs. The use of these non-standardized NRs could be a pitfall in surgical reporting, leading to mix-ups of events or even underreporting of anomalous details. A standardized – preferably electronic – operative report, such as synoptic reporting, could considerably impact the adequacy of reporting [17].

In RIVD, 76.4% of the essential steps were adequately documented. Similar to NR, the lowest adequacy rate in RIVD was the inspection of the liver condition (41.8%), the inspection of the gallbladder condition (58.2%), and the circumferential dissection of the cystic duct and artery (61.0%). Although the latter step was adequately dictated in 61.0%, this adequacy rate is significantly different and almost twice as high compared to the same step in NR. Given the fact that misidentification of anatomical structures is the foremost reason for biliary complications, this improvement in adequacy is an important finding. It is a clear indication that audio in this case would be of greater value than NR for the adequate depiction of this step. Audio can easily be synchronized with intraoperative video recordings, which were also proven to be effective in the adequate description of the operative procedure [1, 7–9]. Two other significant differences were found in the transection of the cystic duct and the removal of the gallbladder from the liver bed. One explanation for this finding is that these steps are so apparent in the course of the operation that they are frequently skipped in RIVD. Both steps were almost 100% reported in NR. As might be expected, copy-pasting prewritten formats to all reports have contributed to the fact that these steps were almost never skipped in NR.

According to the Joint Commission guidelines concerning the record of care, treatment, and services, the operative report should be *“written or dictated upon completion of the operative or other high-risk procedure and before the patient is transferred to the next level of care”* [18]. However, this is often not possible due to other responsibilities of the surgeon or time constraints in the operating room. This method of delayed composition is prone to omission or even incorrect representation of essential information. Despite time until completion was not taken into consideration in this study, it is imaginable that certain aspects of the surgical procedure are not adequately represented in the current NR, yet are adequately addressed in RIVD, such as the circumferential dissection of the cystic duct and artery. These two events might be considered as straightforward in LC and only a means to the goal: the clipping of the cystic duct and artery. However, the dissection phase is often the most precarious stage of LC, and many iatrogenic complications occur at this moment. RIVD offers an advantage by instantly capturing critical information that may not end up being documented in the NR. Due to its real-time nature of documentation, it could also lower inaccuracies in reporting this crucial surgical step. As we demonstrated the comparability of RIVD to NR, we believe that the benefit of RIVD will be more evident when being synchronized with endoscopic video recordings. In our sample, two BDIs occurred (both Strasberg Classification Type A; one lesion to an accessory bile duct and one cystic duct stump leak). In both procedures, a reintervention was performed (ligation of the accessory bile duct and stenting of the cystic duct, respectively). The operators requested access to the video recordings and RIVD of their respective procedures as a means of self-assessment and for educational purposes. In these cases, RIVD was reported to be a helpful tool by the operators in question. After reviewing the recordings of both procedures, the expert panel (JL, AM) concluded that these injuries were unavoidable as the surgical proceedings were according to best practice guidelines. This study was not focused on or powered to detect any BDIs, therefore, no conclusions can be drawn in respect of the occurred BDIs.

During the initial phase of the study setup, some surgeons expressed concerns about RIVD being too intrusive in the operating room. However, with the emergence of technological advancements like the surgical black box, most surgeons recognized the importance of such research in gaining a better understanding of the need for newer types of surgical documentation and identifying current technological limitations. Following the study, surgeons had an overall positive experience. Lastly, operators of one of the participating centers expressed that actively reporting the essential steps of the operation during surgery continually prompted them with memory items. This resembles the crew resource management checklists that are in use in aviation as reminders to ensure that all necessary checks have been completed by the entire crew [19]. As pre- and post-operative checklists have proven to be effective for safe surgery [20], this additional auditory reporting method, in which the operators provide continuous feedback to themselves and the OR personnel, could serve as an intraoperative checklist. The question still remains if this new reporting method could also improve the early detection of operative progress and surgical complications and may even further elucidate unintended deviations from best practice guidelines during surgery.

In the majority of included cases, LC was performed as treatment for symptomatic cholelithiasis. Most procedures were therefore relatively straight-forward cholecystectomies, being an operation with a most reproducible series of operative steps and little variation in outcomes, as evidenced by an operative time of approximately 43 min. Therefore, it is less likely to see any substantial difference between the two methods for recording the operative report compared to even a series of LCs performed for acute cholecystitis where the condition of the gallbladder and the ability to perform a complete LC (rather than a subtotal LC) is decreased. Though, during our analyzes, it was noted in RIVDs of less straight-forward LCs (with a duration of an hour or more) that surgeons were more talkative during the depiction of surgical steps by explaining more context around a surgical step. Although this contextual information did not necessarily improve the adequacy of reporting the surgical steps, it did clarify the surgical proceedings between each essential surgical step. Our expert panel of surgeons has concluded that the audible communication and collaboration between primary surgeon and colleagues during complicated LCs (with anatomic variations or severe bile leakages) were more evident compared to those in which audio was not recorded. It is however important to consider potential biases that could affect the accuracy of verbal or written reports used to obtain CVS, such as the time and manner in which the dictation is conducted. Additionally, the severity and complexity of the case may still influence the reporting of events, as more attention may be required from the surgeon during the operation, potentially affecting RIVD. Considering these potential biases that may exist, we recommend that the operator should always have the option to make additional remarks or add information to the existing recordings in a postoperative report. In most cases, the recordings were made by the first operator, with occasional comments from the second operator. Considering the skill level of the operator (surgeon, fellow, surgical resident), it is possible that their attention might be more focused on the surgical procedure rather than dictation. However, stratifying the data based on the operator's level of experience could introduce complexities due to simultaneous conversations between operators. To ensure an accurate analysis of the recordings, we have chosen not to stratify these groups in the present study. Nevertheless, in future studies, especially those focusing on clinical implementation, the operator's experience and objective assessments of the gallbladder and the liver to assess the severity and complexity of the case can be taken into account as an important factor to consider.

In terms of our recommendation, we foresee that in the mid-long term, NR will be replaced by real-time documented solutions, which incorporate audio, video, and text into a single easy-to-read report. Ideally, this report will be automatically annotated, allowing the surgeon to quickly find relevant sections of the operation. This technology is currently being tested by Dutch researchers with the goal to make it compatible with existing multimedia recording devices.

### Limitations

In this study, operators were not blinded for the intervention. This could have led to the Hawthorne effect in which individuals knowingly or unknowingly modify an aspect of their behavior in response to an observation [21]. Due to this effect, an increase in the operator’s quality of reporting for both RIVD and NR could have been expected. However, in a previous study, the introduction of systematic recordings in laparoscopic colorectal cancer surgery did not have a significant association with operative report adequacy and therefore the amount of bias caused by this knowledge seems negligible [9].

As modern technology is constantly evolving, storing full-length audio recordings can be simple and inexpensive. The added value of recording the entire operative procedure is that these recordings may incorporate possible adverse events that would have been disregarded otherwise. Technical performance data of the operator can be analyzed with these full-length recordings, so that operators can reflect on their own actions and listen to their train of thoughts during critical operative key moments. An important disadvantage of the full-length audio recordings is that the density of convenient information is low, which will lead to laborious review processes for lengthier operative procedures. As audio requires small data storage space, these recordings can be easily synchronized with endoscopic video, making it an inexpensive and useful surgical quality tool. For clinical use, RIVD of key moments might be a solution for more convenient information retrieval of surgical proceedings.

In this study, we focused more on feasibility than practical applicability. Consequently, this was an experimental setting in which the researchers and the participating centers have become acquainted with the new recording techniques, resulting in some technical failures mainly in the first phase – the adjustment period – of the study. These technical failures diminished rapidly as the inclusion period progressed. One researcher (ÖE) was responsible for the storage of the recordings to curtail technical failures. To further increase the user-friendliness of RIVD, a universal solution is needed for the clinical implementation of routine use of audio recording devices in the operating theater. Fortunately, as multimedia devices are increasingly being integrated into new or renovated operating theaters, the recordings of operative procedures can progress with the “touch of a button” which in turn will result in considerably less technical failures. Furthermore, with the surge of artificial intelligence, we expect that audio will have an even more important role in surgical documentation, as the accuracy of automatic and real-time speech-recognition and transcription is rapidly improving. In the Netherlands, these future-proof operating theaters will shortly be the new standard as many hospitals already have new built-in multimedia systems.

To explore the clinical applicability of this new approach, all participating centers, as well as an additional facility, are currently in talks to clinically implement synchronous video recording and RIVD for laparoscopic cholecystectomy, and if feasible, for other types of surgical procedures as well. With this study we aspire to extend our research and enhance RIVD to provide a more robust and user-friendly operative report.

## Conclusion

Real-time intraoperative audio recording is comparable to the postoperatively written operative report in terms of reporting essential surgical steps in LC but demonstrates higher adequacy in reporting essential aspects of the procedure. Audio recording can thus be an important tool for the adequate description of the actions performed during surgery.

### Supplementary Information

Below is the link to the electronic supplementary material.

Supplementary file1 (DOCX 1439 KB)

Supplementary file2 (DOCX 15 KB)

Supplementary file3 (DOCX 20 KB)

## Data Availability

The data that support the findings of this study are included in this published article. Restrictions apply to the availability of the full data sets of the SONAR-trial, which were used under license for the current study, and so are not publicly available.

## Abbreviations

LC: Laparoscopic cholecystectomy
RIVD: Real-time intraoperative voice dictation
NR: Narrative operative report
CVS: Critical view of safety
BDI: Bile duct injury
SONAR: Simultaneous Video and Audio Recording of Laparoscopic Cholecystectomy Procedures

## Declarations

This study was preregistered in the Netherlands Trial Register under number: Trial NL6822 (NTR7008). It was ethically approved by the Medical and Ethical Review Committees of Erasmus University Medical Center, Isala, Zuyderland Medical Center, and IJsselland Hospital.

## Data sharing and data accessibility statement

If requested, deidentified data, collected for the SONAR-trial, the study protocol, and informed consent form can be made available, with exception of the audio recordings, due to privacy policies. Please contact Prof. Dr. Johan F. Lange (j.lange@erasmusmc.nl), who will review all requests with the SONAR-trial investigators. Requests should fulfill the following access criteria: research can only be conducted in collaboration with and after approval of the members of the SONAR-trial investigators, and with a signed data access and sharing agreement. The SONAR-trial investigators must approve all research done with the shared data.
