# Supplementary material for: A comparison between real-time intraoperative voice dictation and the operative report in laparoscopic cholecystectomy: a multicenter prospective observational study
Source: Langenbecks Arch Surg. 2023 Aug 25;408(1):334. doi: 10.1007/s00423-023-03079-w (PMC10457217; doi:10.1007/s00423-023-03079-w)
Supplement: Supplementary file 1 — Supplementary file1 (DOCX 1439 KB) [file 423_2023_3079_MOESM1_ESM.docx]

**Supplementary Information**

**
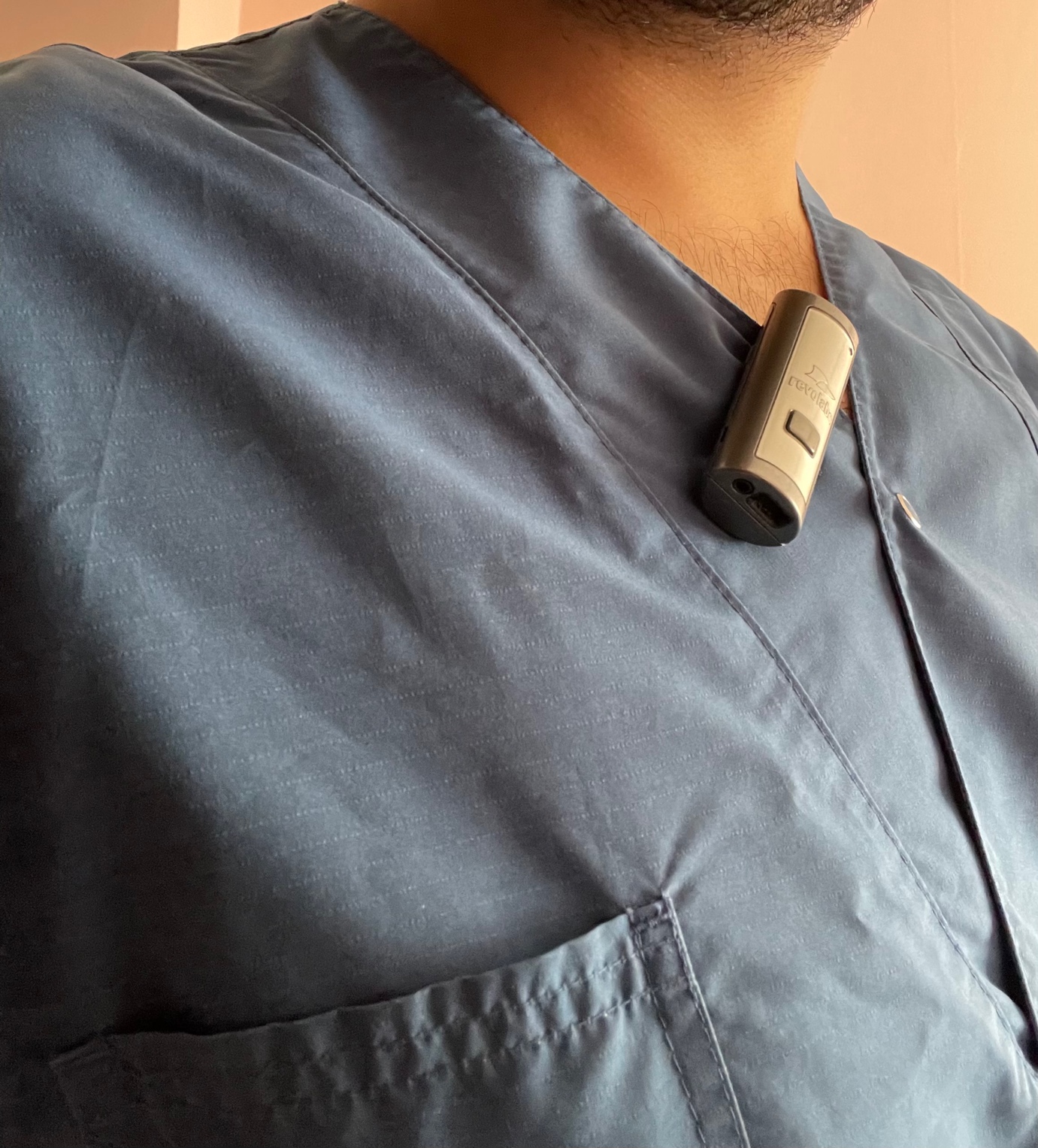
**

**Supplementary image 1**. Wireless microphone attached to the operator's scrub top.

**
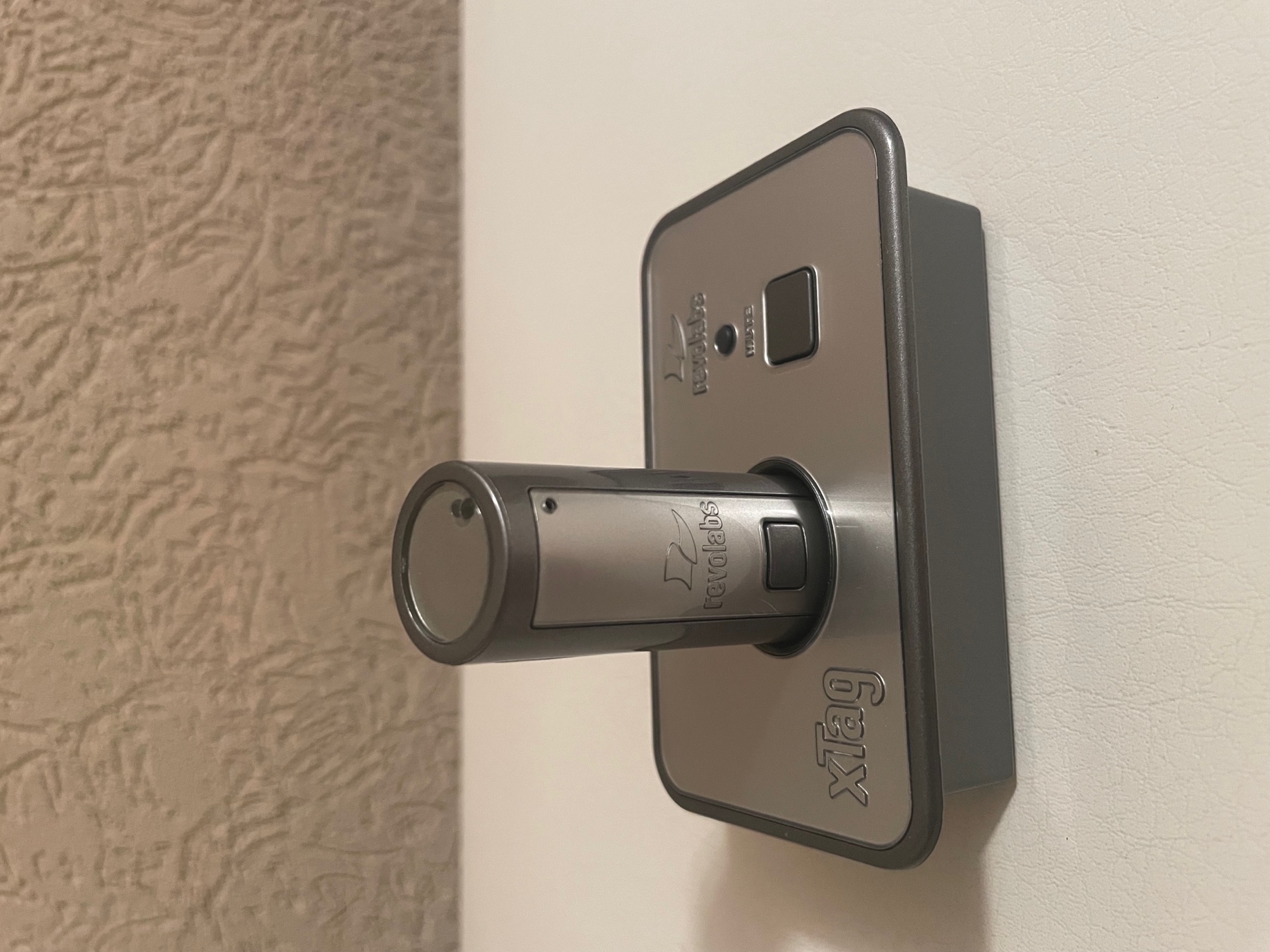
**

**Supplementary image 2**. Wireless microphone placed in its charger base.
