# Supplementary material for: A comparison between real-time intraoperative voice dictation and the operative report in laparoscopic cholecystectomy: a multicenter prospective observational study
Source: Langenbecks Arch Surg. 2023 Aug 25;408(1):334. doi: 10.1007/s00423-023-03079-w (PMC10457217; doi:10.1007/s00423-023-03079-w)
Supplement: Supplementary file 2 — Supplementary file2 (DOCX 15 KB) [file 423_2023_3079_MOESM2_ESM.docx]

# Appendix A

## Requirements for an adequate NR and RIVD.

Based on an evidence-based Dutch guideline: Diagnosis and treatment of cholelithiasis. Association of Surgeons of the Netherlands (NVvH); 2016. [Available from: <https://richtlijnendatabase.nl/richtlijn/galsteenlijden/galsteenlijden_-_startpagina.html>].

**Step 1**: Introduction and positioning of trocars under vision

- 1. Introduction of the first accessory trocar under vision
  2. Introduction of the second accessory trocar under vision
  3. Introduction of the third accessory trocar under vision

**Step 2**: Inspection of surgery site

- 1. Inspection and description of the gallbladder condition
  2. Inspection and description of the liver condition

**Step 3**: Circumferential dissection of the cystic duct and artery

**Step 4**: Transection of the cystic artery (by clipping or sealing)

**Step 5**: Transection of the cystic duct (by clipping or sealing)

**Step 6**: Removal of the gallbladder from the liver bed

**Step 7**: Inspection of liver hemostasis

**Step 8**: Presence of bile or stone spill

**Step 9**: Use of saline irrigation (if used)

**Step 10**: Placement of drain (if present)

**Step 11**: Removal of trocars under vision and check for port side bleeding (intraperitoneal trocar sites)

- 1. Removal of the first accessory trocar under vision
  2. Removal of the second accessory trocar under vision
  3. Removal of the third accessory trocar under vision
