# Supplementary material for: A comparison between real-time intraoperative voice dictation and the operative report in laparoscopic cholecystectomy: a multicenter prospective observational study
Source: Langenbecks Arch Surg. 2023 Aug 25;408(1):334. doi: 10.1007/s00423-023-03079-w (PMC10457217; doi:10.1007/s00423-023-03079-w)
Supplement: Supplementary file 3 — Supplementary file3 (DOCX 20 KB) [file 423_2023_3079_MOESM3_ESM.docx]

**Form 1. Independent reviewer form**

| LAPAROSCOPIC CHOLECYSTECTOMY INDEPENDENT REVIEWER FORM (SONAR-TRIAL) | | | | | | | | | | | | | |
| --- | --- | --- | --- | --- | --- | --- | --- | --- | --- | --- | --- | --- | --- |
|  | |  |  |  | | | | | | | | | |
| ANONYMIZED Case iDENTIFICATION CODE | | | | | \|  \|  \|  \|  \|  \|  \|  \|  \|  \|  \|  \| \| --- \| --- \| --- \| --- \| --- \| --- \| --- \| --- \| --- \| --- \| --- \| | | | | | | |  |  |
| REVIEW DATE | | | | | \| D \| D \| M \| M \| Y \| Y \| Y \| Y \| \| --- \| --- \| --- \| --- \| --- \| --- \| --- \| --- \| | | | | | |  |  |  |
| Reviewer (initials, last name) | | | | | \|  \| \| --- \| | | | | | | |  |  |
|  | |  |  |  | | | | | | | | | |
| STEPS RECORDED | | | | | | | | | | | | | |
|  | | | | | | NR | | RIVD | | COMMENTS | | |  |
| **1a** | Introduction of the first accessory trocar | | | | | Yes | No | Yes | No |  | | |  |
|  |  |  |  |  |  |  |  |  |  |  | | |  |
| **1b** | Introduction of the second accessory trocar | | | | | Yes | No | Yes | No |  | | |  |
|  |  |  |  |  |  |  |  |  |  |  | | |  |
| **1c** | Introduction of the third accessory trocar | | | | | Yes | No | Yes | No |  | | |  |
|  |  |  |  |  |  |  |  |  |  |  | | |  |
| **2a** | Inspection of the gallbladder | | | | | Yes | No | Yes | No |  | | |  |
|  |  |  |  |  |  |  |  |  |  |  | | |  |
| **2b** | Inspection of the liver condition | | | | | Yes | No | Yes | No |  | | |  |
|  |  |  |  |  |  |  |  |  |  |  | | |  |
| **3** | Circumferential dissection of the cystic duct and artery | | | | | Yes | No | Yes | No |  | | |  |
|  |  |  |  |  |  |  |  |  |  |  | | |  |
| **4** | Transection of the cystic artery | | | | | Yes | No | Yes | No |  | | |  |
|  |  |  |  |  |  |  |  |  |  |  | | |  |
| **5** | Transection of the cystic duct | | | | | Yes | No | Yes | No |  | | |  |
|  |  |  |  |  |  |  |  |  |  |  | | |  |
| **6** | Removal of the gallbladder from the liver bed | | | | | Yes | No | Yes | No |  | | |  |
|  |  |  |  |  |  |  |  |  |  |  | | |  |
| **7** | Inspection of liver hemostasis | | | | | Yes | No | Yes | No |  | | |  |
|  |  |  |  |  |  |  |  |  |  |  | | |  |
| **8** | Presence of spill (clear or purulent bile, stones) | | | | | Yes | No | Yes | No |  | | |  |
|  |  |  |  |  |  |  |  |  |  |  | | |  |
| **9** | Saline irrigation (if used) | | | | | Yes | No | Yes | No |  | | |  |
|  |  |  |  |  |  |  |  |  |  |  | | |  |
| **10** | Drain placement (if present) | | | | | Yes | No | Yes | No |  | | |  |
|  |  |  |  |  |  |  |  |  |  |  | | |  |
| **11a** | Removal of the first accessory trocar | | | | | Yes | No | Yes | No |  | | |  |
|  |  |  |  |  |  |  |  |  |  |  | | |  |
| **11b** | Removal of the second accessory trocar | | | | | Yes | No | Yes | No |  | | |  |
|  |  |  |  |  |  |  |  |  |  |  | | |  |
| **11c** | Removal of the third accessory trocar | | | | | Yes | No | Yes | No |  | | |  |
|  |  |  |  |  |  |  |  |  |  |  | | |  |
